# Research on the construction of a digital medical consumables asset management system based on information ecology theory

**DOI:** 10.3389/fdgth.2026.1946134

**Published:** 2026-10-13

**Authors:** Qi Wan, Yifan Yang, Meihua Cai, Ke Wang, Kai He

**Affiliations:** Asset Management Department, Shanghai Key Laboratory of Maternal Fetal Medicine, Shanghai Institute of Maternal-Fetal Medicine and Gynecologic Oncology, Shanghai First Maternity and Infant Hospital, School of Medicine, Tongji University, Shanghai, China

**Keywords:** asset management, digital technology, information ecology theory, life-cycle management, medical consumables

## Abstract

Medical consumables constitute a core object of hospital asset management, and the traditional extensive management model can no longer meet the requirements of refined management in modern hospitals. This narrative review aims to construct a conceptual theoretical framework based on information ecology theory, to analyze the patterns of information flow in the field of medical consumables management, and build a digital asset management ecosystem centered on the four elements of “information platform–digital technology–responsible actor–social environment”. As a narrative review, it synthesizes selected literature and policy documents rather than providing a systematic or exhaustive synthesis of evidence. The conceptual contribution of this framework is to address the “technology-heavy, system-light” limitation of existing research by shifting toward ecological governance through the coordination of the four elements, and proposing an operating mechanism in which the information chain links the closed loop of asset management. The framework is illustrated through a worked example of high-value consumable use during cesarean section. The framework is illustrated through a worked example of high-value consumable use during cesarean section. It provides a conceptual reference and a hypothesis-generating roadmap for future research and local adaptation, rather than a validated operational solution. All thresholds, targets, automated responses, and organizational arrangements mentioned in this article are proposed design principles for future validation, not empirically confirmed standards.

## 1 Introduction

The quality of medical consumables management is directly related to medical safety and operational efficiency. With the deepening of China's medical and health system reform, the National Health Commission has issued a series of documents, including the Administrative Measures for Medical Consumables in Medical Institutions (Trial) (1) and the Action Plan for Promoting the High-Quality Development of Public Hospitals (2021–2025) (2), explicitly requiring medical institutions to achieve informatization and sustainable development in consumables management. Although progress has been made in cost reduction, whole-process traceability, and compliant use, several problems remain widespread in management practice, such as “emphasizing procurement while neglecting maintenance”, “emphasizing use while neglecting efficiency”, and “emphasizing clinical practice while neglecting management”. These problems have led to improper resource allocation, low utilization efficiency, increased safety hazards, and rising operating costs (3). As a result, current practice can hardly adapt to the new healthcare reform landscape, and it is urgent to introduce new technical means and management theories to achieve systematic restructuring.

In recent years, digital technologies represented by the Internet of Things (IoT) (4), radio-frequency identification (RFID) (5), cloud computing (6), machine learning (7), and blockchain (8) have evolved rapidly, offering a feasible pathway of “whole-process empowerment” for medical consumables management. Unique Device Identification (UDI) enables life-cycle traceability through “one item, one code”; RFID smart cabinets enable real-time inventory perception and dynamic replenishment; machine learning models such as Multimodal Temporal Networks (MTN) support demand forecasting and inventory optimization; and blockchain guarantees the tamper-resistance of procurement, use, and settlement data. Internationally, in July 2024 the European Union issued Commission Implementing Decision (EU) 2024/2120, extending the designation period of UDI issuing entities to 2029 (9). According to the EU Medical Device Regulation (MDR), from May 2026 all newly marketed medical devices must be registered in the UDI and Device Registration modules (10). In 2024, Thai scholars proposed a third-party-logistics-based SPD supply chain network model and used the AHP–Fuzzy TOPSIS method to systematically evaluate hospital supply chain modes (11). Commercial market reports estimate that the global medical consumables management system market was valued at USD 5.247 billion in 2024 and is projected to reach USD 7.842 billion by 2031 (12), while the global medical device SPD market was valued at approximately USD 14.98 billion in 2024 and is projected to reach USD 31.98 billion by 2031 (13). These figures are non-peer-reviewed commercial projections and are cited only to indicate market activity and expected growth, not as evidence of effectiveness or clinical benefit.

However, existing research still focuses mainly on the application effects of individual technologies and lacks a systematic examination of the collaborative mechanisms in medical consumables asset management. Fragmented problems—such as difficulties in post-deployment system integration, low clinical acceptance, and data security risks—remain prominent. Specifically, current research shows evident gaps in three respects: (1) inadequate research on the collaborative mechanisms among various digital technologies, as UDI, RFID, SPD, and machine learning systems are often built independently, with heterogeneous data standards and mutually incompatible interfaces; (2) insufficient research on information coordination among multiple organizational actors (clinical departments, management departments, suppliers, and information technology departments), with ambiguously defined information ecological niches and missing collaborative mechanisms; and (3) an as-yet-unfilled theoretical gap in incorporating policy-environment variables (such as mandatory UDI implementation, Diagnosis-Related Groups (DRG)/DIP (Disease Diagnosis-Intervention Packet) payment reform, and centralized volume-based procurement) into system design. This “technology-heavy, system-light” orientation makes it difficult for technology investment to translate into substantive improvements in management performance.

Information ecology theory provides systematic theoretical support for constructing a collaborative medical consumables management system (14). In contrast to the traditional “instrumental rationality”perspective, this theory holds that information, technology, organizational actors, and the social environment should be regarded as an interdependent, dynamically co-evolving ecosystem. In the field of medical and health information management, the information ecology perspective has been used to analyze the operational mechanisms of electronic health record systems and the organizational embedding of personal health record systems, and, in the management of online health information service quality, information ecology theory has also been used to analyze, from a systems perspective, the pathways and effects of information resource utilization. Introducing information ecology theory into digital medical consumables management not only implies the adoption of advanced technical means but also constitutes a fundamental reshaping of the governance paradigm.

Compared with theoretical frameworks commonly used in existing research on medical consumables management, information ecology theory has distinctive explanatory advantages. Supply chain management theory (SCM) focuses on logistics efficiency and cost optimization but pays insufficient attention to the multi-directional interaction of information flow and the constraining role of the institutional environment (15). The Technology Acceptance Model (TAM) focuses on individual users' acceptance of technology but fails to cover organizational-level issues such as multi-actor collaboration and systemic ecological evolution (16). The Task–Technology Fit (TTF) model emphasizes the fit between technology characteristics and task requirements but overlooks the dynamic influence of external variables such as the policy environment (17). Resource dependence theory focuses on organizational reliance on external resources but does not incorporate information as a core resource into its analytical framework (18). In contrast, information ecology theory integrates the four elements of information, technology, actors, and environment into a unified analytical framework, emphasizing dynamic coordination and co-evolution among the elements. It is therefore more suitable for explaining the complex scenarios of medical consumables management, which involve multiple actors, technologies, and institutions intertwined.

Adopting information ecology theory as the analytical framework, this paper narrative review synthesizes selected literature domestic and international research progress and practical applications in digital medical consumables asset management, summarizes typical technological pathways and management modes, identifies the shortcomings and challenges of existing research, and proposes a construction scheme for a digital asset management system based on information ecology theory. The aim is to provide a theoretical reference and practical guidance for the asset management transformation of medical institutions, particularly consumables-intensive departments such as obstetrics and gynecology.

### 1.1 Concepts and methods

#### 1.1.1 Terminology and scope

In this article, “medical consumables” refers to disposable or limited-use medical devices used in clinical diagnosis and treatment that are approved by the drug regulatory authority and are not capitalized as fixed assets. This category includes high-value consumables, low-value consumables, implants, and *in vitro* diagnostic reagents. “Medical devices” is used as the broader regulatory category, which includes both consumables and durable equipment. “Medical equipment” refers to reusable, durable, capital-intensive devices and systems, such as imaging systems, monitors, and reusable surgical instruments; it is not the primary object of this review and is discussed only where it is relevant to the information chain of consumables management. UDI requirements apply to defined regulatory categories of medical devices, including certain consumables, rather than to every type of hospital consumable. The scope of this article is therefore limited to medical consumables as defined above. Evidence drawn from equipment or medication management is treated as adjacent evidence rather than direct evidence for consumables management.

#### 1.1.2 Review approach and scope

This narrative review aims to develop a theory driven conceptual framework. The sources actually cited in this review were selected purposively according to their thematic relevance to the four information-ecology elements—information, technology, actors, and environment—and to the core technologies and policy changes discussed in the manuscript, including UDI/GS1, RFID, SPD, machine learning, cloud computing, blockchain, DRG/DIP payment reform, and centralized volume-based procurement. The cited materials comprise: (i) peer-reviewed journal articles in English and Chinese, covering UDI traceability, RFID inventory management, SPD supply-chain models, machine-learning demand forecasting, cloud computing, and blockchain in healthcare; (ii) official policy, regulatory, and standards documents issued by Chinese and international authorities, such as the National Health Commission, the National Medical Products Administration, the National Healthcare Security Administration, the European Commission, the U.S. FDA, ANMAT, and the Turkish Ministry of Health; (iii) authoritative market and industry reports, such as those from QYResearch, Global Info Research, and Zebra Technologies; and (iv) selected authoritative website/news sources and maturity-model documentation, such as HIMSS EMRAM and The Beijing News. The cited materials span from foundational information-ecology work (Nardi & O’Day, 2000) to policy documents and studies published up to March 2026. Literature and documents were identified through purposive selection, citation chaining, and the authors' domain expertise. No systematic search protocol, PRISMA flow diagram, record counts, formal eligibility screening, or risk-of-bias/GRADE appraisal was applied, because the review is narrative and conceptual in nature. Accordingly, the cited evidence is illustrative and interpretive rather than exhaustive, and the proposed indicators, mechanisms, and framework are design principles for future empirical validation, not empirically confirmed standards.

#### 1.1.3 Evidence hierarchy and use of non-peer-reviewed sources

This narrative review draws on heterogeneous sources. For claims about effectiveness or management outcomes, priority is given to peer-reviewed studies, with systematic reviews and controlled or pre-post studies ranked highest. Official policy, regulatory, and standards documents are used to establish legal and policy context, not to demonstrate effectiveness. Commercial market reports, industry surveys, news reports, and maturity-model documents are used only as contextual or descriptive sources. They are not treated as equivalent to independent peer-reviewed evidence. Where such sources are cited, their non-peer-reviewed status and commercial or institutional interests are stated in the text and table notes.

## 2 Information ecology theory and its applicability to medical consumables asset management

Information ecology theory is an interdisciplinary theory formed by introducing the theories and methods of natural ecology into the information domain. Nardi et al. (19) argued that the systems view of information ecology theory embeds information activities in a dynamic environment jointly constituted by technology, people, practices, and values. Chinese scholars have localized this theory, generally holding that the information ecosystem involves four elements—information, people, technology, and environment (20)—which do not simply add up but form a dynamic, co-evolutionary ecosystem.

Medical consumables asset management involves multiple links such as procurement, inventory, use, billing, and traceability, and encompasses many actors including hospital asset management departments, clinical departments, suppliers, information departments, and regulatory agencies; it therefore essentially constitutes a typical information ecosystem. As shown in Table 1, the specific mapping of the four elements in consumables management is as follows:

**Table 1:** Core connotations of information ecology theory and their conceptual mapping to consumables management.

Element	Connotation	Corresponding aspect in consumables management	Current prominent issues
Information	“Energy flow” of the system; fundamental resource for decision-making	Purchase orders, inventory data, usage records, billing information, etc	Information silos, inconsistent data
Technology	Intermediary tools for information collection, transmission, and processing	RFID, UDI, cloud computing, machine learning, blockchain	Difficult system integration, inconsistent interfaces
Humans	Producers, transmitters, and consumers of information	Asset department, clinical department, supplier, IT department, regulatory agency	Weak collaboration, unclear responsibilities
Environment	Macro constraints such as systems, policies, and culture	UDI policy, centralized procurement DRG/DIP, Information Security Regulations	Lagging system adaptation

However, from the perspective of current system operation, the collaborative awareness among actors in medical consumables asset management remains weak, responsibilities and rights are ill-defined, interests are imbalanced, information lag is widespread, and collaborative regulatory mechanisms are still imperfect, so a well-functioning closed-loop management has not been achieved. This indicates that the information ecosystem is still at a developmental stage, and the coordination among the four elements urgently needs to be enhanced. Information ecology theory prompts a shift in medical consumables asset management from “the application of a single technological tool” to “platform-based governance through multi-element collaboration”, providing a systematic framework for constructing an efficient, safe, and compliant modern hospital management system. By introducing this theory, we can not only address the current fragmentation but also promote the transformation of the management model toward intelligence and systematization, laying a solid foundation for building an efficient, safe, and compliant modern hospital management system.

## 3 Current application of digital technologies in medical consumables asset management

The application of digital technologies in medical consumables asset management has moved from single-point pilots to systemic integration, forming a technology chain of “identification and traceability–intelligent perception–intensive management–predictive analytics–collaborative security” (see Figure 1). Table 2 summarizes the technical characteristics of the six core technologies, and the following description is limited to empirical evidence related to the four dimensions that make up this article.

**Figure 1:**
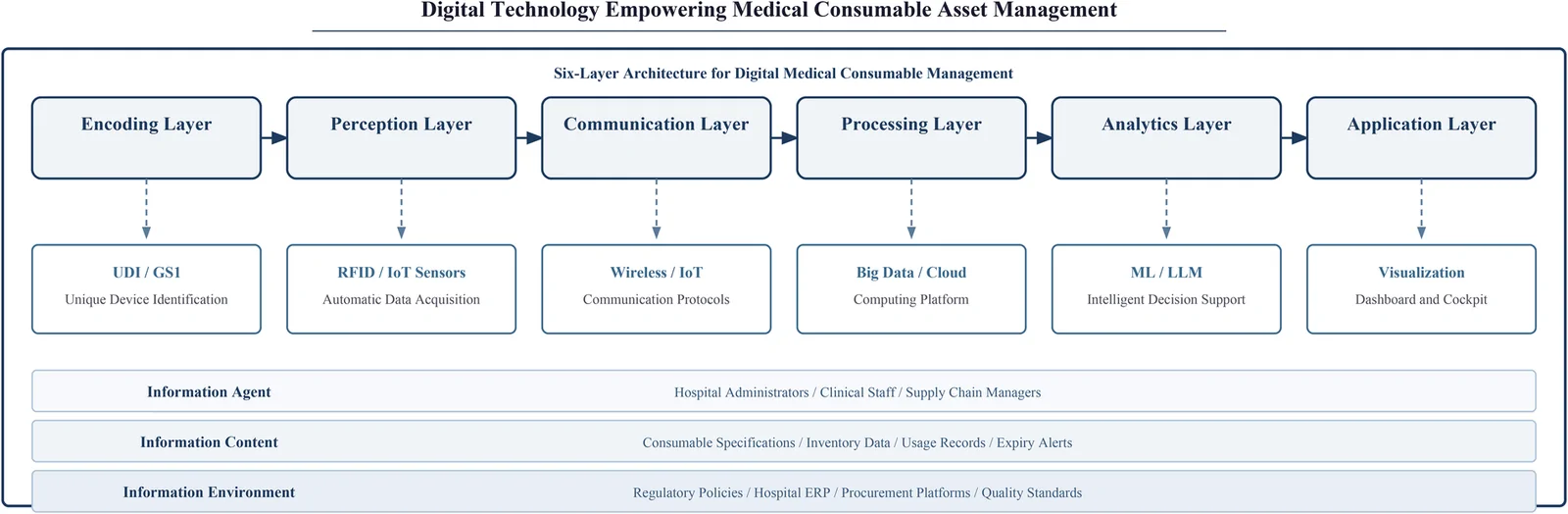
Six-layer digital architecture for medical consumables asset management based on information ecology theory.

**Table 2:** Summary of six digital technologies in medical consumables asset management.

Technology	Core function in consumables management	Illustrative evidence	Dimension chiefly addressed	Principal limitation
UDI/GS1	Unique identification enabling “one item, one code” life-cycle traceability	Analysis of 1,979,655 registered UDIs against national claim codes (Republic of Korea) (21); pre–post comparison of 7,745 vs. 7,932 consumable records (China) (22)	Technology integration; data governance	Coverage limited to defined regulatory categories; depends on coding compliance
RFID	Contactless batch identification; smart cabinets for real-time inventory sensing	Intraoperative medication inventory implementation (5); vendor-sponsored survey of 280 hospital decision-makers (23)	Technology integration; organizational acceptance	Tag detachment and mis-scans; much cited evidence concerns equipment, not consumables (24, 25)
SPD mode	Outsourced supply–processing–distribution with third-party logistics	AHP–fuzzy TOPSIS evaluation of network models (Thailand) (11); order and distribution analysis at a tertiary Class-A hospital (Guangzhou) (26)	Technology integration; institutional support	Data-security exposure; dependence on supplier capability (27)
Machine learning	Demand forecasting and inventory optimization	LSTM/ICSL forecasting on multi-city real-world data (28); multimodal temporal network reducing forecasting error to 6.3% (7)	Technology integration (decision layer)	Requires high-quality inputs; little consumables-specific external validation
Cloud computing	Unified data centre integrating HIS, SPD, and operational data	Public-hospital operational large model: management response speed improved by 95%, exception warning response <5 min (29)	Technology integration; data governance	Security and compliance obligations (30); single-site report
Blockchain	Tamper-resistant traceability and cross-institutional trust	Systematic reviews of blockchain-enabled medical supply chains (31, 32); UDI–blockchain traceability pilots (33)	Data governance	Throughput, storage cost, and difficulty of interfacing with legacy systems

Peer-reviewed studies, official policy documents, commercial market reports, industry surveys, and news sources are not equivalent. Market reports and industry surveys are used only for contextual purposes and are not treated as independent evidence of effectiveness. Where evidence is derived from equipment or medication settings, it is labelled as adjacent evidence.

### 3.1 Identification and traceability: UDI and RFID

UDI is the cornerstone for life-cycle management of medical consumables. The U.S. FDA issued the final UDI rule in 2013, and the EU has mandated UDI implementation under the MDR. According to Announcement No. 21 of 2026 issued by the National Medical Products Administration, the National Health Commission, and the National Healthcare Security Administration (34), from 1 June 2027 all Class II medical devices (including *in vitro* diagnostic reagents) and Class I *in vitro* diagnostic reagents must bear unique device identifiers. The application pathway can be summarized as “three-code unification and four-code interconnection”—integrating the medical insurance code, traceability code, material code, and consumables product code—operationalized through “scan-in, scan-out, scan-use, and scan-settle”. The GS1 standard underpins most implementations; Shanghai piloted medical product traceability from 2007 with 120 participating hospitals (35, 36), and GS1-based traceability has since been mandated by regulators in other jurisdictions including Argentina (37) and Türkiye (38).

Two studies provide the strongest empirical support. Choi et al. (21) evaluated post-market surveillance in the Republic of Korea and found that among 1,979,655 registered UDIs only 36.02% were classified as insurance-covered items, and among 42,171 claim codes only 32.62% had registered UDIs, averaging 53 UDIs per claim code; this many-to-many mapping conflates safe and unsafe device models and reduces signal-detection sensitivity, demonstrating the value of UDI for real-world evidence generation and precision regulation. Duan Legang et al. (22) compared 7,745 consumable records under traditional management with 7,932 under a UDI-based closed-loop model, reporting significantly better dispensing efficiency, inventory compliance, timely supply, and dispensing standardization (*P* < 0.05), with reduced waste and adverse events.

RFID complements UDI by enabling contactless, batch identification: smart consumables cabinets sense placement and removal in real time, handheld readers replace item-by-item counting, and reading at each circulation node builds an electronic traceability chain (5). However, some studies have shown (24, 25) that RFID is used on reusable medical devices rather than disposable consumables; It is included because the recognition mechanism is directly transferred, but the difference in accuracy between the original research scope is worth noting. Evidence specific to consumables remains thin: the most frequently cited source is a survey of 280 non-clinical decision-makers at large hospitals in the United States and United Kingdom, in which 84% considered modernization of asset and inventory management necessary and roughly 70% planned RFID or real-time locating deployment within five years (23); this survey was industry-sponsored and measured stated intentions rather than outcomes, so it is treated here as suggestive contextual evidence of demand rather than as proof of effectiveness.

### 3.2 Supply–processing–distribution (SPD) mode

The SPD mode is an intensive management model grounded in supply chain management theory and supported by information technology (39). It integrates internal hospital resources with third-party supply chain resources so that consumables are dispatched directly from suppliers and distribution is outsourced to professional logistics providers, freeing storage space and reducing hospital labor costs; it couples this with dynamic “one item, one code” tracking, automated stocktaking, billing and invoice matching, expiration alerts and threshold-based replenishment; and it shifts settlement from a “procure-first-then-use” to a demand-driven “use-first-then-consume” pattern, which in turn requires stronger supplier quality assessment.

Empirically, Thai scholars used AHP–fuzzy TOPSIS to evaluate third-party-logistics-operated SPD network models (11); a study at a tertiary Class-A hospital in Guangzhou analyzed order and distribution data for 10 categories of high-value consumables from 2023 through the first half of 2024 and confirmed improved distribution response and procurement efficiency (26); and a further study reported strengthened supervision of consumables and *in vitro* diagnostic reagents and reduced operating costs, while cautioning about data-security risks (27). Commercial market analyses project rapid SPD growth (40). But these numbers come from different reports using different market definitions and are reported only as indicative, non peer reviewed background.

### 3.3 Intelligent decision support: machine learning

Demand-forecasting models based on big data and machine learning address the high volatility of consumables demand by combining historical consumption, surgical scheduling, and external environmental variables. Zhang Lei et al. (41) applied K-means clustering and support vector machines to improve the efficiency of high-value consumables use; Liang Min et al. (42) introduced deep learning and kernel-based graph-clustering to improve information-mining accuracy; and Liu Bin et al. (43) added a consumables life-cycle AI module to a hospital resource planning system, extracting procurement, inventory, and use data to support procurement and supplier decisions. For time-series demand, long short-term memory networks outperformed traditional regression and other neural models on real-world multi-city data, with a follow-up ICSL algorithm improving accuracy by nearly 30% (28); the multimodal temporal network proposed by Zhao Zhiyuan integrates historical consumption, surgical scheduling, and external events and reduces forecasting error to 6.3%, outperforming ARIMA (7). These results are promising but derive largely from emergency-supply or general-supply settings rather than from routine hospital consumables management.

### 3.4 Infrastructure and trust: cloud computing and blockchain

Cloud computing provides the unified infrastructure on which the above technologies depend, integrating procurement, warehousing, use, and settlement data through centralized storage and distributed processing (30), and enabling interconnection of the HIS, SPD system, and operational data center within a single data center. A news report described a single-site, vendor-reported implementation of a large-scale model for public-hospital operational management in Beijing, in which management response speed was reported to improve by 95% and exception-warning response was shortened to under five minutes (29). This source is a non-peer-reviewed news report, and the figures are vendor-reported and independently unverified. It is cited only as an illustrative example of current market activity, not as evidence of effectiveness.

Blockchain uses cryptographic algorithms to make records tamper-resistant, transparent, and traceable (44), addressing privacy leakage, data tampering, and information silos in medical data management (45). The IBM Food Trust platform and the MediLedger project illustrate whole-process traceability in food and pharmaceutical supply chains (46); domestically, the Information Center of the National Medical Products Administration has explored blockchain-based UDI traceability, and some institutions have combined blockchain with UDI for whole-process monitoring of high-value consumables (33). Systematic reviews confirm that blockchain performs particularly well for traceability and has moved from proof-of-concept to systematic evaluation (31, 32).

### 3.5 Synthesis: what the evidence supports across the four dimensions

Read against the four dimensions, the evidence base is markedly uneven. Technology integration is the best supported: UDI, SPD, and cloud platforms each have studies reporting improved traceability, distribution efficiency, or response times (21, 22, 26, 29), though almost all are single-institution pre–post or descriptive designs without concurrent controls. Organizational acceptance is the weakest: the principal source is a vendor-sponsored survey of stated intentions (23), supplemented by anecdotal reports that staff revert to manual logging after smart-cabinet deployment; in the literature selected for this narrative review, we did not identify any controlled study of clinical adoption of consumables scanning. Data governance shows demonstrated technical feasibility—blockchain traceability and tiered storage (32, 33)—but no evidence on governance outcomes such as breach rates or data-quality trajectories in consumables settings. Institutional support has essentially no primary evidence; claims rest on policy documents and expert commentary rather than evaluation.

This asymmetry is itself the warrant for the present paper. The six technologies do not operate in isolation but form a mutually supporting ecosystem (Figure 2): UDI and RFID provide the data foundation, SPD builds the business-process framework, machine learning drives decision support, cloud computing supplies computing power, and blockchain underwrites information security. Yet the literature evaluates them one at a time and overwhelmingly along a single dimension. What is missing is not another single-technology study but an analytical framework capable of holding all four dimensions together.

**Figure 2:**
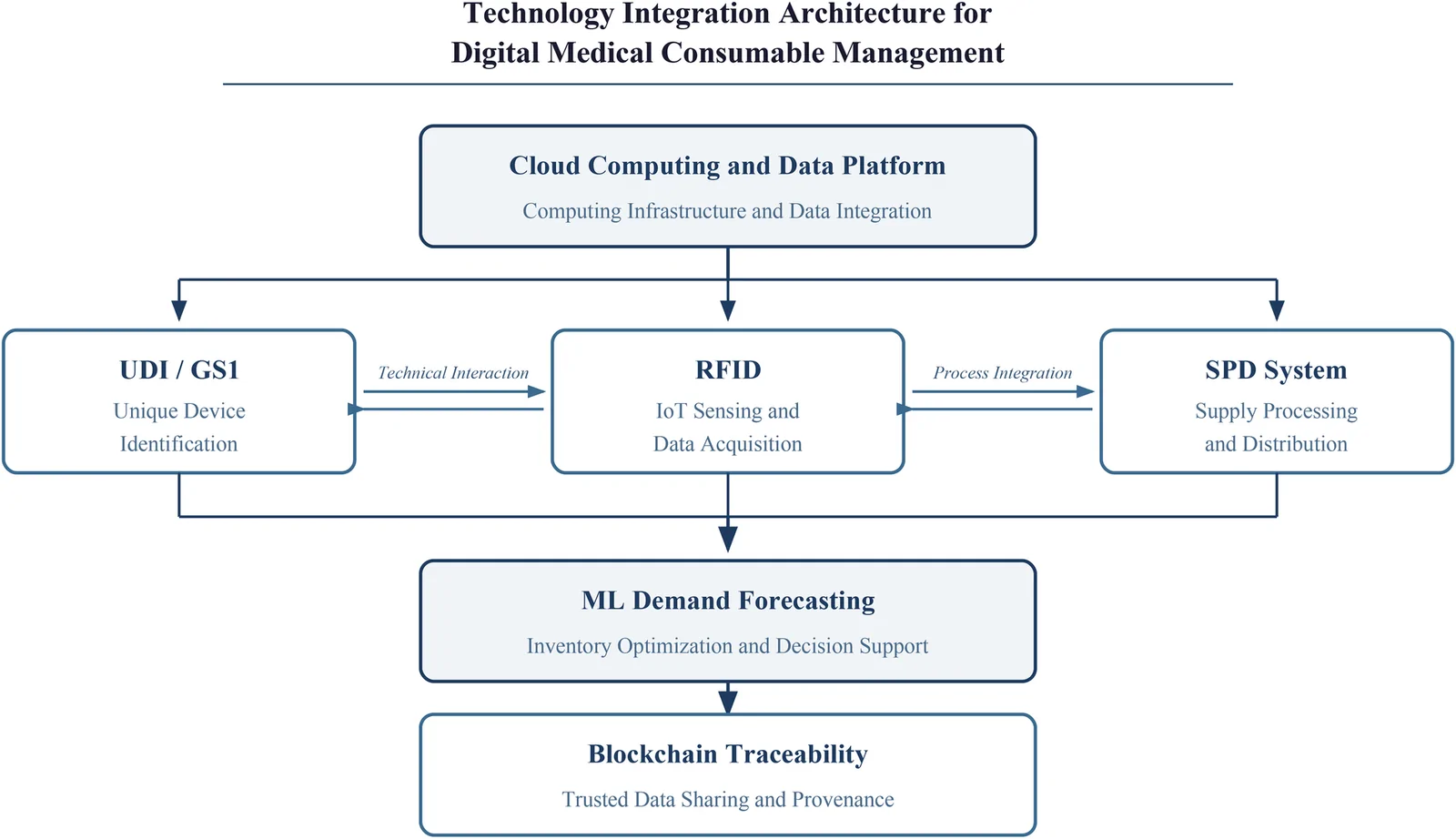
Technology integration architecture for digital medical consumables management.

## 4 Construction of a digital medical consumables asset management system based on information ecology theory

### 4.1 Construction rationale

Digital technologies have achieved partial results in medical consumables management. However, current practice exhibits pronounced “technology-stacking” characteristics: each system is built independently, data standards are heterogeneous, and interfaces are mutually incompatible, so that the potential benefits of the technologies are not fully realized. For instance, RFID smart cabinets can accurately record dispensing but cannot automatically synchronize with the financial system; machine learning models can predict demand but lose timeliness because procurement processes rely on manual approval. This phenomenon reveals the limitation of technology-centric thinking: technology itself does not necessarily lead to improved management performance; the key lies in whether the technology can be embedded in an overall framework of information flow, actor collaboration, and institutional adaptation.

Information ecology theory provides theoretical guidance in this respect. The theory emphasizes that the successful operation of any information system requires dynamic balance and coordinated evolution among the four elements of information, technology, actors, and environment. When applied to medical consumables management, this requires a shift from the instrumentalist mindset of “purchase a software suite and deploy several pieces of equipment” to the construction of an ecosystem in which the four elements are in dynamic balance. On this basis, and using information ecology theory as the framework, this chapter proposes the overall design of a digital medical consumables asset management system.

### 4.2 Overall framework

Before presenting the system design, it should be emphasized that the specific thresholds, indicators, workflows, and organizational arrangements described below are illustrative design principles derived from the proposed framework. They are not empirically validated standards. Their appropriateness, feasibility, and exact values should be determined through local needs assessment, pilot implementation, and prospective evaluation. Where policy requirements are cited, they are identified as such; all other parameters should be regarded as proposed design options rather than confirmed benchmarks.

The core logic of this system can be summarized as “one objective, four types of elements, and three linked chains”. “One objective” refers to closed-loop management of the entire life cycle of medical consumables assets that is “safe, efficient, traceable, and low-cost” (see Figure 3). “Four types of elements” refers to the information platform, digital technology, responsible actors, and social environment—these are not independent modules but interdependent, dynamically evolving components of an ecosystem. “Three linked chains” refers to the information chain linking and traversing the industrial chain, the risk chain, and the responsibility chain, so that the flow of materials, risk alerts, and accountability tracing operate in a coordinated manner.

**Figure 3:**
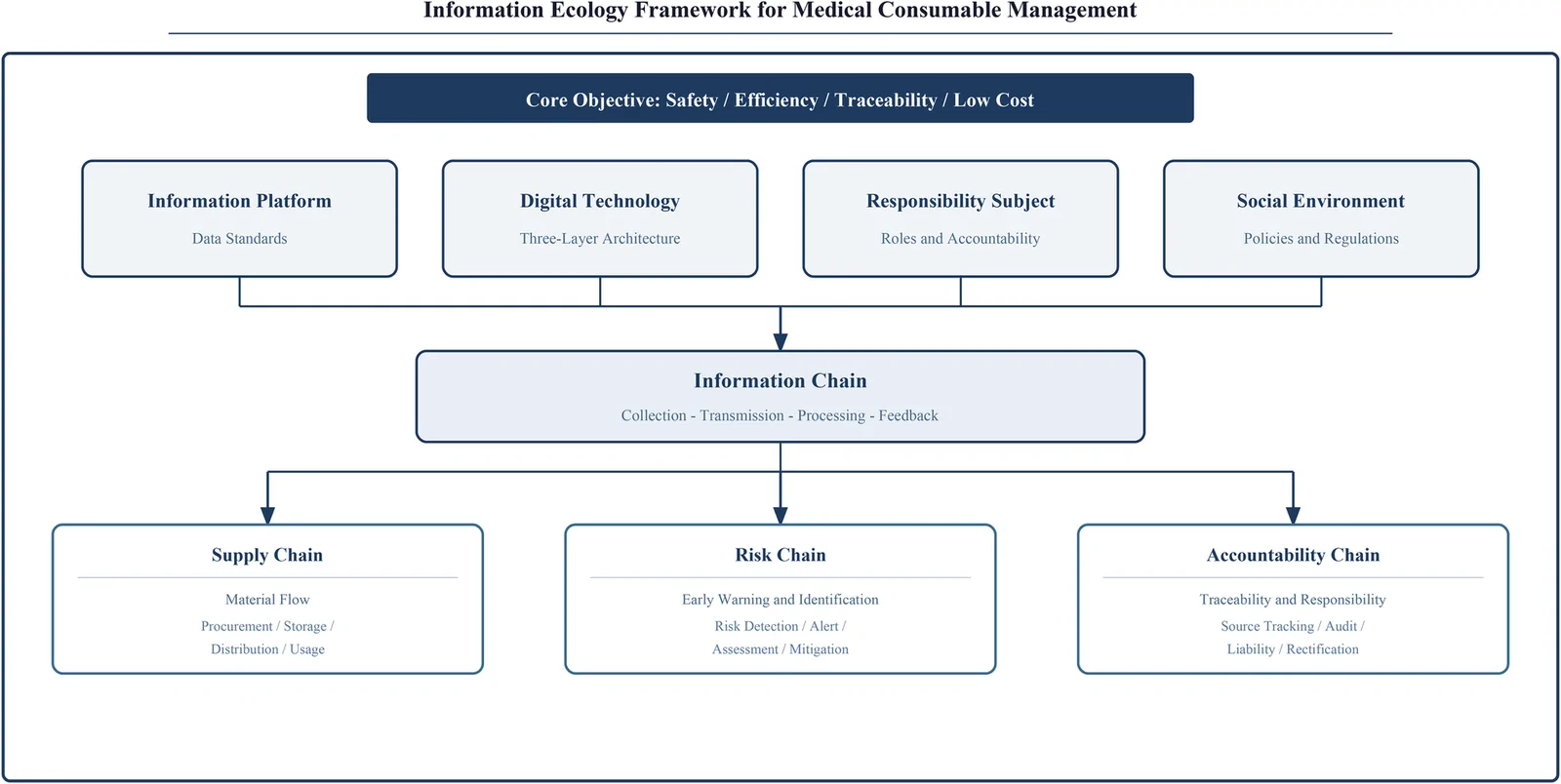
Information ecology framework for medical consumables management.

Specifically, the information chain comprises four steps: “acquisition–transmission–processing–feedback”. When the information chain is fully connected, it can link upstream suppliers, midstream hospital warehouses and clinical departments, and downstream patients into a visualized industrial chain; it can embed risk-identification mechanisms at each node to form an alert chain covering supply risk, inventory risk, use risk, and billing risk; and it can clearly record the responsible actor at each operation node, thereby constructing a traceable and attributable responsibility chain. Once the information chain is broken, the industrial chain becomes uncontrollable, the risk chain develops blind spots, and the responsibility chain becomes blurred. Therefore, the primary task of system construction is to ensure the full connectivity of the information chain.

### 4.3 Design of core elements

#### 4.3.1 Information platform

Information is the “energy source” of the ecosystem. Building a unified information platform requires accomplishing three tasks. First, code unification: fully implementing UDI/GS1 standards and, in accordance with National Medical Products Administration Announcement No. 21 of 2026 (34), achieving “one item, one code” connectivity from consumables factory dispatch to patient use. Second, master data management: establishing a basic consumables information repository covering codes, names, specifications, manufacturers, suppliers, and prices to enable “one-code universal lookup”. Third, a data middle-platform: integrating HIS, SPD, HRP, and financial systems to synchronize procurement orders, inventory dynamics, use consumption, and billing settlement in real time. A data-accuracy target such as ≥99.5%≥99.5% is proposed here only as an illustrative design parameter, not as an empirically validated standard; the appropriate threshold should be calibrated locally and evaluated prospectively.

#### 4.3.2 Digital technology

Technology is the intermediary tool for information acquisition, transmission, and processing. Building on the technology overview above, this paper proposes a three-layer technology architecture of “perception layer–middle-platform layer–application layer” (see Table 3).

**Table 3:** Three-layer architecture of digital technologies.

Layer	Technical composition	Core functionality	Correlation with information ecology
Perception layer	UDI/RFID tags, smart shelves, handheld terminals, temperature/ humidity sensors	Real-time collection of consumables location, quantity, expiration date, and usage status	Ensure timeliness and accuracy of information collection
Middle platform layer	Cloud computing, big data platform, edge gateway	Data cleaning, storage, standardization, breaking down heterogeneous systems barriers	Enables centralized processing and cross-system information flow
Application layer	Machine learning, blockchain, visual cockpit	Demand forecasting, inventory optimization, automatic replenishment, tamper-proof traceability, decision support	Transforms information into intelligent decision-making

#### 4.3.3 Responsible actors

Actors are the producers, transmitters, and consumers of information, and each occupies a different “information ecological niche”. The asset management department/medical engineering department is the core management actor, responsible for procurement planning, inventory scheduling, and supplier management; clinical departments are the primary users and data sources, and the standardization of their scanning operations directly affects the quality of information acquisition; suppliers are the supply actors, required to deliver on time, code items in a standardized manner, and achieve system integration; the information department provides technical support and system maintenance; and the regulatory agency is responsible for policy guidance and compliance supervision. These five categories of actors are not connected by hierarchical command relationships but by a collaborative network based on information sharing. To support collaborative efficiency, hospitals may consider, as proposed organizational design options, establishing a periodic joint conference mechanism, incorporating indicators such as leakage rate, expiration loss rate, and traceability completeness into departmental performance assessment, and using blockchain-based rules to trigger alerts for non-compliance and support accountability tracing. These arrangements are conceptual recommendations requiring local adaptation and empirical evaluation; they are not validated standards. Any automated alert or accountability action should be subject to institutional policy, data-governance review, and pilot validation.

#### 4.3.4 Social environment

The social environment constitutes the institutional constraints and external drivers of the system. The most pressing policy changes at present include: the mandatory implementation of UDI (fully in effect by June 2027); supply-stability pressures brought about by centralized volume-based procurement; the DRG/DIP payment reform, which links consumables costs directly to disease-group profit and loss; and the compliance requirements for medical data imposed by the Personal Information Protection Law and the Data Security Law. These environmental factors are not static backdrops but variables to which the system must dynamically adapt. For example, the dynamic adjustment of centralized procurement catalogs requires that the system be capable of supplier evaluation and rapid switching; DRG/DIP payment requires that consumables-use data be linkable to the grouping system to enable real-time cost measurement. A mature digital management system should be able to perceive changes in the environment and automatically adjust operating parameters.

### 4.4 Operating mechanism

The core of this system's operation lies in the smooth flow of the information chain. The information chain consists of four steps—acquisition, transmission, processing, and feedback—linking the industrial chain, the risk chain, and the responsibility chain in sequence. Obstetric surgery is a suitable setting in which to make this concrete, because cesarean section combines high procedure volume with a dense and largely disposable consumable set, and because the organization of that consumable set has measurable operational consequences: in a recent medico-economic and environmental evaluation at a university hospital, standardizing cesarean-section consumables into pre-assembled kits reduced median operative set-up time by 165.5 s and yielded documented annual savings of €8,011.08, with the largest benefit during emergency procedures (47). The following walkthrough traces a single high-value item—an absorbable hemostatic sealant used during an elective cesarean section—through the four steps. It is a conceptual illustration of expected system behavior, not a report of an implemented or empirically evaluated system.

1. Acquisition: Before a scheduled cesarean section, a labor and delivery nurse retrieves the hemostatic sealant from an RFID smart cabinet in the obstetric operating suite, and the system automatically reads the tag information (name, batch number, expiry date, and unique code) and records the retriever, the time, and the associated patient ID.
2. Transmission: The data are uploaded in real time to the cloud middle-platform via the intra-hospital 5G/IoT network, simultaneously triggering the HIS billing linked to the cesarean-section surgical record and the corresponding DRG/DIP diagnosis-related group, eliminate manual-entry errors.
3. Processing: The middle-platform runs a machine learning model that, based on the obstetric department's surgical schedule and historical consumption data, predicts whether inventory can meet demand over the next three days; if the level falls below a locally defined safety threshold, the system is designed, as a proposed design principle, to generate a purchase order and send it to the supplier's system, subject to institutional approval rules and pilot validation. At the same time, blockchain records the use event, forming a tamper-resistant traceability chain.
4. Feedback: If a consumable is within an illustrative pre-expiration window (e.g., 30 days, to be calibrated locally), the system is designed to send an alert to the medical engineering department and the obstetrics department; if billing records do not match the actual quantity dispensed for the cesarean section, the system is designed to flag the discrepancy and notify the audit department. Any automated hold or suspension of requisition privileges via smart-contract-style rules is a proposed, policy-dependent design option, not an established or validated mechanism; it should be implemented only after institutional authorization and pilot validation. Managers can view real-time consumables-flow status hospital-wide through the management cockpit.

Through this closed loop, the system achieves real-time coordination of the industrial chain (on-demand replenishment by suppliers), proactive alerts of the risk chain (stockouts, expirations, and violations), and precise tracing of the responsibility chain. The example also shows how the four dimensions bind together in a single workflow: the acquisition and transmission steps are feasible only where systems are integrated (technology integration); they depend on a nurse scanning reliably under time pressure in an operating suite (organizational acceptance); the resulting patient-linked records must be protected and quality-assured (data governance); and the billing linkage to DRG/DIP grouping only functions where payment policy and internal accountability rules support it (institutional support). A failure in any one dimension breaks the chain regardless of the sophistication of the others.

### 4.5 Theoretical comparison with existing management models

To further clarify the theoretical positioning and unique value of the proposed framework, it is necessary to conceptually compare it with several theoretical frameworks commonly used in medical information management. See Table 4.

**Table 4:** Comparison of the proposed framework with existing theoretical and practice-oriented models.

Framework	Explanatory strength	Limitation for consumables asset management/contrast with this framework
Technology Acceptance Model (TAM) (16)	User adoption of information systems via perceived usefulness and ease of use	Unit of analysis is the individual; cannot explain multi-actor collaboration or institutional change. This framework treats five actor categories as a collaborative network.
Task–Technology Fit (TTF) (17)	Fit between technology characteristics and task requirements	Overlooks policy environment, organizational culture, and inter-system interoperability. This framework treats the social environment as an endogenous element.
Supply chain management theory (15)	Allocation of logistics, capital, and information flows across supply nodes	Focuses on efficiency and cost of the “chain”, not the multi-directional “network” or ecosystem evolution. This framework adds risk warning and accountability traceability.
Resource dependence theory (18)	How organizations manage dependence on external resources	Does not treat information as a core strategic resource, nor explain information value creation under digitalization.
Resource-Based View/dynamic capabilities	Competitive advantage through integrating and reconfiguring resources	Oriented to the internal organization; limited attention to external institutions and collaboration networks.
HIMSS EMRAM (48)	Eight-stage maturity assessment of hospital electronic medical record capability	Concentrates on clinical information functions; little attention to materials management or supply chain coordination.
Information ecology applied to CSSD–OR consumables (49)	Three-tier framework of information integration, technology adaptation, and institutional alignment at the sterile-supply/operating-room interface	Closest prior work and the direct antecedent of this paper. Scope is a single inter-departmental interface; the present framework extends to the hospital-wide asset life cycle and to external policy variables (UDI, DRG/DIP, centralized procurement).

In summary, the framework elevates “information” to the core energy of the system, positions “technology” as the intermediary tool of information flow, incorporates multiple “actors” into a collaborative network rather than a hierarchy, and transforms “environment” from an external constraint into an endogenous system variable. This analytical framework seeks to extend and integrate insights from existing theories in terms of unit of analysis (individual versus system), temporal dimension (static fit versus dynamic evolution), and spatial scope (intra-organizational versus ecosystem), providing a candidate, as-yet-empirically-untested, theoretical perspective for understanding and guiding the digital transformation of medical consumables asset management.

### 4.6 System positioning

Compared with the traditional experience-based management mode and single-technology application schemes, the core distinction of the proposed system lies in a shift of focus. Traditional management focuses on inventory quantities, single-technology applications focus on functional implementation, while the proposed system focuses on the coordinated relationships among information, technology, actors, and environment. In terms of information flow, traditional management is fragmented and delayed, single-technology applications can achieve only local connectivity, whereas the proposed system pursues whole-chain closed-loop flow. In terms of actor relationships, traditional management is siloed with unclear rights and responsibilities; in single-technology applications, personnel often passively adapt to the system; whereas the proposed system emphasizes multi-actor collaboration and clear rights and responsibilities. In terms of environmental responsiveness, traditional management passively copes with policy changes, single-technology solutions rarely consider institutional adaptation, whereas the proposed system internalizes environmental variables as constituent components of the system design.

This system positioning determines its applicability boundaries: for tertiary hospitals with complex consumables categories and high requirements for management granularity, especially obstetrics and gynecology hospitals, the proposed system offers substantial reference value; for small-scale medical institutions with a single consumables category and simple management needs, the system can be tailored and implemented in accordance with practical circumstances.

## 5 Challenges and countermeasures

### 5.1 Barriers to technology integration

Current hospital informatization construction exhibits typical “phased” characteristics: the HIS system may be procured from Vendor A, the SPD system from Vendor B, and the financial system developed by Company C. Different systems have disparate data standards, interface protocols, and communication formats, leading to widespread “inability to connect and align” problems. For example, the whole-chain traceability rate of high-value consumables from suppliers to patients at a tertiary Class-A hospital in China is less than 40% (7); the root cause is not the failure of RFID or UDI technology itself, but rather that data cannot flow automatically between systems—procurement information resides in the supplier's system, inbound records in the SPD system, and use billing in the HIS system, and the three must be manually exported and imported to form a complete chain. Such “system silos” expose the technology-based closed loop to the risk of breaking at the very first link.

The key response to this challenge is to establish a standardized data-exchange system. Hospitals should not adopt a radical “tear-down-and-rebuild” strategy; instead, they should build a data middle-platform or integration platform that, without replacing existing systems, provides unified data interfaces and conversion rules across systems. Specifically, hospitals can refer to medical information exchange standards such as HL7 and FHIR and, combined with the UDI coding system, formulate intra-hospital consumables data-exchange specifications; middleware can be used to enable data extraction, cleaning, and synchronization across heterogeneous systems. When Sun Yat-sen Memorial Hospital of Sun Yat-sen University procured a medical-consumables intelligent-agent platform, it explicitly required the system to be capable of interfacing with multiple systems including HIS, SPD, and LIS, which is an embodiment of an integration mindset. In addition, hospitals should require newly procured consumables-management systems to provide standard APIs and incorporate interface openness and data interoperability into supplier evaluation criteria.

### 5.2 Low clinical acceptance

The value of a technology system ultimately depends on user acceptance. In medical consumables management, clinical staff are the key nodes for information acquisition—operations such as scanning at dispensing, checking expiration dates, and confirming billing must be completed by them. However, frontline personnel are generally in a state of “time poverty”: surgeries, ward rounds, and medical-record documentation already occupy their working time, and any additional operational procedures may trigger resistance. Some hospitals have found, after deploying RFID smart cabinets, that some nurses continue to use traditional manual logging, causing deviations between system data and actual conditions. Even more challenging, elderly medical staff are less familiar with smart devices, and mis-scans and missed scans occur from time to time, directly affecting the integrity of the information chain.

Addressing this problem requires both technology adaptation and management incentives. At the technology level, “ultra-simple operation” should be pursued: smart cabinets should support batch retrieval with automatic identification, eliminating item-by-item scanning; handheld terminals should use voice prompts and icon guidance to reduce reading burden; and the system should automatically link patient information to avoid duplicate entry. At the management level, consumables management indicators (such as scan-completeness rate and expiration-date review rate) should be incorporated into departmental performance assessment, and dedicated incentives should be established; simultaneously, tiered training should be conducted with one-on-one mentoring for older staff and “seed users” cultivated among young talents to promote adoption. Notably, the cultivation of operational discipline requires continuous positive feedback: once clinical departments see that the system can automatically replenish stock and reduce stockout waiting times, acceptance will increase naturally.

### 5.3 Data security and quality risks

Another challenge brought about by digital management is data security and quality. Consumables data contain sensitive information such as patient identities and treatment information, as well as commercial secrets such as hospital procurement prices and supplier relationships. Once data are leaked or tampered with, the consequences are severe. Although blockchain provides tamper-resistance, it is not a panacea—its performance bottlenecks (transaction throughput), storage costs, and difficulty of interfacing with traditional systems make it difficult to fully replace centralized databases in the short term. In addition, data quality issues cannot be ignored: non-standard UDI coding, RFID tag detachment, and manual-entry errors can lead to “garbage in, garbage out”, undermining the trustworthiness of machine learning model predictions.

In response to data governance challenges, a tiered protection and closed-loop quality mechanism should be established. On the security side, classified and hierarchical data management should be implemented: consumables-use data involving patient privacy should be stored and transmitted with encryption, and access rights should be granted on the principle of least necessity; core traceability data (such as implantation records of high-value consumables) can be recorded on the blockchain for evidentiary purposes, while routine inventory data can remain in traditional databases for efficiency. On the quality side, a data-quality inspection regime should be established: the system should automatically detect the integrity of UDI codes, the success rate of RFID reads, and the consistency between billing and dispensing, raising real-time alerts for anomalies and tracing them to responsible individuals. In addition, periodic data cleaning should be conducted to remove redundant, erroneous, or outdated information and ensure the accuracy of middle-platform data.

### 5.4 Institutional lag and talent shortage

The continuous operation of a digital management system depends on institutional support. Two salient problems are currently faced. First, poor policy coordination. The mandatory implementation of the UDI system has entered the countdown, but some hospitals are still in a wait-and-see mode, lacking a clear timeline and clear assignment of responsibility; the integration of centralized procurement policies with the SPD mode is not yet mature, and standards for supplier data integration have not been unified; the DRG/DIP payment reform requires real-time cost measurement of consumables, but in most hospitals the financial system and the consumables-management system have not yet been interoperated. Second, a shortage of interdisciplinary talent. Digital management of consumables requires “medical engineering + IT + management” interdisciplinary personnel who understand the characteristics of clinical consumables, are familiar with information technology, and are skilled in supply chain management, yet such positions are almost vacant in current hospital staffing, and existing personnel are largely transitioned from traditional warehouse managers, making it difficult for them to undertake data analysis and system operation and maintenance.

Resolving the institutional dilemma requires combining external compliance drivers with internal capacity building. Hospitals should establish a consumables digital management working group led by hospital leadership, incorporating tasks such as UDI implementation, system integration, and data governance into the annual priority agenda, with clearly defined responsible departments and time nodes, and reporting progress to the hospital administrative meeting on a regular basis. In terms of talent, an “external recruitment plus internal cultivation” strategy can be adopted: hospitals can recruit professionals from medical informatization enterprises to serve as data administrators, while dispatching medical engineering staff to training in data analysis, supply chain management, and other areas; hospitals can also cooperate with universities to establish tailored training programs to build a talent reserve. In addition, hospitals should formulate “Regulations for Digital Management of Medical Consumables” that specify operating norms, data standards, and penalties for violations for each actor, so that the management system operates in accordance with rules.

### 5.5 Applicability and limitations of the theoretical framework

Information ecology theory provides a systematic analytical framework for this study; however, when applied to medical consumables asset management, it also has certain inherent limitations that must be acknowledged in both theoretical understanding and practical application.

First, tension between the theory's macro-orientation and its operationalization. Information ecology theory originates from a philosophical observation of the ecosystems of the information society, and its core concepts (such as “information ecological niche”, “co-evolution”, and “energy flow”) have strong metaphorical color and macro-explanatory power. However, translating these concepts into operable management indicators and executable implementation paths in specific management scenarios still requires substantial intermediate theoretical construction and empirical validation. For example, how can “the smoothness of the information chain” be quantitatively assessed? How can “dynamic balance among the four elements” be monitored and warned? Answering these questions requires the further development of specific assessment tools and measurement systems.

Second, the causal mechanisms and interaction weights among the four elements remain unclear. Information ecology theory emphasizes the mutual dependence and coordinated evolution of information, technology, actors, and environment, but it does not clearly explain the direction of causal relationships, the intensity of interactions, or the weight distribution across contexts. In the specific practice of medical consumables management, does technological change drive changes in actor behavior, or does institutional-environment change compel technological upgrades? The dominant role of each element may vary substantially across contexts, and information ecology theory currently lacks refined mechanisms to explain such differences.

Third, the applicability of the theory to small medical institutions requires adjustment. The proposed framework is based on management scenarios of tertiary Class-A hospitals (especially consumables-intensive specialty hospitals such as obstetrics and gynecology hospitals), and its four-element structure and three-chain operating mechanism presuppose a certain organizational scale, technology base, and management capability. For small medical institutions with a single consumables category, simple management processes, and a weak informatization base, directly applying this framework may lead to “over-engineering”—i.e., system design that is too complex, with implementation costs exceeding actual benefits. Such institutions should tailor and simplify the framework according to their circumstances, focusing on the most urgent tasks of information-chain connectivity and basic data governance.

Fourth, the empirical basis of information ecology theory needs to be strengthened. Although the theoretical framework has logical self-consistency and explanatory power, empirical studies of information ecology theory in the social and medical fields remain relatively limited. Existing research consists mostly of conceptual elaboration or case description and lacks large-scale quantitative validation and cross-institutional comparative research. The validity, reliability, and universality of the proposed framework still need to be tested and refined through subsequent empirical methods such as multi-case studies, questionnaire surveys, and maturity evaluations.

Fifth, insufficient attention to the influence of power structures and conflicts of interest on the information ecosystem. Information ecology theory tends to describe the information ecosystem as a relatively harmonious and spontaneously evolving organic whole, but it pays insufficient attention to power asymmetry, conflicts of interest, and political dynamics among actors in the system. In medical consumables management, structural tensions exist between clinical departments pursuing diagnostic and therapeutic convenience and asset departments pursuing cost control, and between hospitals seeking low-price procurement and suppliers pursuing profit maximization. How these tensions shape the direction, quality, and integrity of information flow is a direction in which the theoretical framework needs to be deepened further.

In summary, information ecology theory provides an inspiring analytical perspective for digital asset management of medical consumables, but its application requires a clear theoretical stance—it is an “analytical framework” rather than an “operations manual”; its value lies in providing systemic thinking and an ecological perspective, not in replacing specific implementation details and locally adapted institutional design. Future research should, on the basis of this framework, continually enrich, revise, and refine the theoretical connotations through empirical studies, and thereby advance its evolution from a “conceptual framework” to an “operable theoretical model”.

## 6 Conclusions

This study is conceptual and hypothesis-generating. The framework proposed here is derived from information ecology theory and a purposive narrative review; it has not been empirically validated as an operational solution and should be read as a candidate analytical framework for future testing, local adaptation, and refinement, not as a confirmed management standard or implementation blueprint. All thresholds, targets, automated responses, and organizational arrangements mentioned in this article are proposed design principles for future validation, not empirically confirmed standards.

Grounded in information ecology theory, this review examines the current and potential application of UDI, RFID, SPD, machine learning, cloud computing, and blockchain in medical consumables asset management. It proposes a conceptual digital management framework centered on four interdependent elements—information platform, digital technology, responsible actors, and social environment—and outlines an operating mechanism in which the information chain links the industrial chain, the risk chain, and the responsibility chain. Throughout, the review focuses on medical consumables as defined in Section 1.1. UDI-related statements are limited to consumables within UDI-regulated medical device categories, and evidence from medical equipment or medication management is treated as adjacent evidence rather than direct evidence for consumables management.

The evidence base is uneven and should be interpreted accordingly. In line with the evidence hierarchy stated in Section 1.2, peer-reviewed studies are prioritized for claims about effectiveness or management outcomes; official policy, regulatory, and standards documents are used to establish policy context rather than to demonstrate effectiveness; and commercial market reports, industry surveys, news reports, and maturity-model documents are used only as contextual or descriptive sources. Such non-peer-reviewed sources are not treated as equivalent to independent peer-reviewed evidence. Commercial market projections are not relied upon in this review because different providers use different market definitions and their figures are not directly comparable; where commercial activity is mentioned, it is presented only as background.

The framework inherits several limitations of information ecology theory. First, core concepts such as “information ecological niche,” “co-evolution,” and “energy flow” are metaphorically rich but difficult to operationalize into measurable indicators. Second, the causal direction, interaction intensity, and weight distribution among the four elements remain unclear and may vary across contexts. Third, the framework presupposes a certain organizational scale, technology base, and management capacity, and may be less applicable to small institutions with simple consumables categories and weak informatization. Fourth, empirical validation of information ecology theory in medical consumables management remains limited; existing evidence is mostly conceptual or case-based, lacking large-scale quantitative and cross-institutional comparative studies.

Fifth, the theory pays insufficient attention to power asymmetries and conflicts of interest among actors, such as tensions between clinical departments pursuing convenience and asset departments pursuing cost control, or between hospitals seeking low-price procurement and suppliers pursuing profit maximization. These limitations reinforce the need to treat the framework as conceptual and hypothesis-generating rather than as an empirically confirmed operational model.

The theoretical contribution of this study is to apply information ecology theory as a conceptual lens to medical consumables asset management. We do not claim that this is the first such application or that the framework transcends existing theories. Its contribution is to extend and integrate existing perspectives—including TAM, TTF, supply chain management theory, resource dependence theory, RBV, and EMRAM—by treating information, technology, actors, and environment as interdependent elements and by proposing an information-chain mechanism that links the industrial, risk, and responsibility chains. Its practical value is to offer a candidate conceptual framework and a set of hypothesis-generating design principles for future empirical validation, not a validated operational solution.

The following recommendations are conceptual, hypothesis-generating design principles derived from the proposed framework. They are not empirically validated standards, implementation protocols, or performance benchmarks. They should be adapted, piloted, and prospectively evaluated locally before any routine adoption.

### 6.1 Recommendations for hospital administrators

First, strengthen organizational safeguards by establishing a medical consumables digital management working group led by responsible hospital leaders and integrating the asset management or medical engineering department, information center, clinical departments, and finance department into a unified coordination mechanism. Second, adopt phased implementation: standardize UDI coding and master data for consumables within UDI-regulated categories, deploy RFID smart cabinets and SPD systems for inventory management, and then introduce machine learning forecasting and blockchain traceability. Third, incorporate consumables management indicators such as scan-completeness rate, near-expiration loss rate, book-to-actual match rate, and traceability completeness into departmental assessment and link them to performance-based pay. These recommendations are proposed for future testing and local adaptation; they are not validated operational requirements.

### 6.2 Policy recommendations for policymakers

First, accelerate the formulation of nationally unified data-exchange standards for medical consumables, clarifying API interface standards, data dictionaries, and transmission protocols. Second, establish a regional-level collaborative data platform for consumables management to enable cross-institutional visualization and alerts. Third, promote real-time linkage between the DRG/DIP payment system and the consumables-management system so that consumables cost data can be uploaded and linked to settlement, allowing clinical departments to view the impact of consumables consumption on disease-group profit and loss. These recommendations are also proposed for future testing and local adaptation; they are not validated operational requirements.

Future research should prioritize empirical validation of the proposed framework through multi-case studies, questionnaire surveys, maturity evaluations, and controlled or quasi-experimental designs. It should also explore the application of emerging technologies such as large language models and digital twins. The framework's value lies in generating testable propositions and guiding local adaptation, not in providing a ready-to-implement operational solution.
